# Supplementary material for: Comparing retinotopic maps of children and adults reveals a late-stage change in how V1 samples the visual field
Source: Nat Commun. 2023 Mar 21;14:1561. doi: 10.1038/s41467-023-37280-8 (PMC10030632; doi:10.1038/s41467-023-37280-8)
Supplement: Supplementary file 3 — Reporting Summary [file 41467_2023_37280_MOESM3_ESM.pdf]

Corresponding author(s): Marc Himmelberg
Last updated by author(s): Feb15,2023

Reporting Summary

Nature Portfolio wishes to improve the reproducibility of the work that we publish. This form provides structure for consistency and transparency in reporting. For further information on Nature Portfolio policies, see our Editorial Policies and the Editorial Policy Checklist.

Statistics

For all statistical analyses, confirm that the following items are present in the figure legend, table legend, main text, or Methods section.

n/a	Confirmed	
☐	☒	The exact sample size (*n*) for each experimental group/condition, given as a discrete number and unit of measurement
☐	☒	A statement on whether measurements were taken from distinct samples or whether the same sample was measured repeatedly
☐	☒	The statistical test(s) used AND whether they are one- or two-sided<br>*Only common tests should be described solely by name; describe more complex techniques in the Methods section.*
☒	☐	A description of all covariates tested
☒	☐	A description of any assumptions or corrections, such as tests of normality and adjustment for multiple comparisons
☐	☒	A full description of the statistical parameters including central tendency (e.g. means) or other basic estimates (e.g. regression coefficient) AND variation (e.g. standard deviation) or associated estimates of uncertainty (e.g. confidence intervals)
☐	☒	For null hypothesis testing, the test statistic (e.g. *F*, *t*, *r*) with confidence intervals, effect sizes, degrees of freedom and *P* value noted<br>*Give P values as exact values whenever suitable.*
☒	☐	For Bayesian analysis, information on the choice of priors and Markov chain Monte Carlo settings
☒	☐	For hierarchical and complex designs, identification of the appropriate level for tests and full reporting of outcomes
☐	☒	Estimates of effect sizes (e.g. Cohen's *d*, Pearson's *r*), indicating how they were calculated

Our web collection on statistics for biologists contains articles on many of the points above.

Software and code

Policy information about availability of computer code

Data collection	fMRI stimuli were presented using MATLAB 2017A, the Psychophysics Toolbox V3 (http://psychtoolbox.org/), and the opensource vistadisp toolbox (https://github.com/vistalab/vistasoft).
Data analysis	fMRI data were preprocessed using fMRIprep v20.0.1 and additional analyses were completed using custom MATLAB code, the Neuropythy V0.11.9 python package (https://github.com/noahbenson/neuropythy), and a customised version of the vistasoft toolbox v1 (https:// vistalab.stanford.edu/software/) . Anatomical MRI data were processed using mrQ software (https://,github.com/,mezera/,mrQ). Custom code is available at https://osf.io/2yqwe/

For manuscripts utilizing custom algorithms or software that are central to the research but not yet described in published literature, software must be made available to editors and reviewers. We strongly encourage code deposition in a community repository (e.g. GitHub). See the Nature Portfolio guidelines for submitting code & software for further information.

Data

Policy information about availability of data

All manuscripts must include a data availability statement. This statement should provide the following information, where applicable:

- Accession codes, unique identifiers, or web links for publicly available datasets
- A description of any restrictions on data availability
- For clinical datasets or third party data, please ensure that the statement adheres to our policy

Source data are provided with this paper. Raw data used in this study were taken from previous work (Gomez et al. 2018, Nature Communications, doi: 10.1038/

s41467-018-03166-3) and will be made available in BIDS format on OpenNeuro upon publication.

Human research participants

Policy information about studies involving human research participants and Sex and Gender in Research.

Reporting on sex and gender	We refer to biological sex in our participant sample. Neither sex nor gender were considered in our study design as this is not relevant to the purpose of our study and there are no sex or gender differences in our data
Population characteristics	The purpose of our study was to compare brain structure of two groups that differed in age (adults 18+, and children 5-12 years). See above.
Recruitment	Participants were recruited via word of mouth in the Bay area. All participants had 'normal or corrected to normal vision' thus any self-selecting bias such an interest in vision due to vision problems was not a confound.
Ethics oversight	Institutional Review Board of Stanford University

Note that full information on the approval of the study protocol must also be provided in the manuscript.

Field-specific reporting

Please select the one below that is the best fit for your research. If you are not sure, read the appropriate sections before making your selection.

☐ Life sciences ☒ Behavioural & social sciences ☐ Ecological, evolutionary & environmental sciences

For a reference copy of the document with all sections, see nature.com/documents/nr-reporting-summary-flat.pdf

Behavioural & social sciences study design

All studies must disclose on these points even when the disclosure is negative.

Study description	Quantitative between-subjects design
Research sample	Adults included members of the Stanford University community (undergraduates, masters students, PhD students, post-docs, 11 females, 13 males, mean age = 23.8 years. Children included members of the Bay area community (11 males, 14 females, mean age = 8.8 years) All participants had normal vision and no history of neurological or psychiatric illness. The study assessed human vision which does not greatly vary key characteristics (i.e., age, gender, socioeconomic status, etc) thus this sample is representative typical vision across the the population and is considered an appropriate study sample.<br><br>The dataset came from the following paper: https://doi.org/10.1038/s41467-018-03166-3
Sampling strategy	We sampled from the participant pool at convenience and availability without regard to gender, or handedness. We aimed for 15 participants based on the sample size of prior studies conducting fMRI measurements of retinotopy (https://doi.org/10.7554/eLife.67685, https://doi.org/10.1016/j.neuroimage.2021.118609, https://doi.org/10.1038/s41467-022-31041-9)
Data collection	Anatomical and functional MRI data were acquired on a 3-Tesla GE Discovery MR750 scanner (GE Medical Systems). Anatomical data were collected using a 32-channel head coil and functional data were collected using a16-channel head coil.<br><br>During adult data collection, the participant and the researcher were present. During child data collection, the participant, their parent, and the researcher were present. All participants were blinded to the research hypotheses during data collection. The experimenters were not blind to the hypothesis.
Timing	Data were collected between 1st May 2014 and 21 November 2016
Data exclusions	Two adults were excluded from the analysis as the retinotopic maps were so poor quality that we could not identify V1 thus we did not use the data. These data are illustrated in supplementary materials.
Non-participation	No participants declined or dropped out of the study
Randomization	Participants were not randomized into experimental groups - we used a between-subjects design in which we compared brain surface of two groups - adults vs children. Controlling of covariates is not relevant to this work; any covariates among our sample (other than age) do not have influence on human vision and would not affect the outcome of this experiment.

Reporting for specific materials, systems and methods

We require information from authors about some types of materials, experimental systems and methods used in many studies. Here, indicate whether each material, system or method listed is relevant to your study. If you are not sure if a list item applies to your research, read the appropriate section before selecting a response.

Materials & experimental systems

n/a	Involved in the study	
☒	☐	Antibodies
☒	☐	Eukaryotic cell lines
☒	☐	Palaeontology and archaeology
☒	☐	Animals and other organisms
☒	☐	Clinical data
☒	☐	Dual use research of concern

Methods

n/a	Involved in the study	
☒	☐	ChIP-seq
☒	☐	Flow cytometry
☐	☒	MRI-based neuroimaging

Magnetic resonance imaging

Experimental design

Design type	Population receptive field mapping
Design specifications	Each participant completed 1 functional session that consisted of 4 fMRI scans. Each scan lasted 6 minutes. Each participant also completed 1 anatomical session consisting of 1 MRI scan. The scan lasted 10 minutes.
Behavioral performance measures	Button press; participants pressed a button when fixation spaceship changed colour. The task is used to ensure that the participant is awake during the task and maintaining fixation. The % correct identification of the fixation cross changing colour was used to assess that the participant was awake and fixation and is of otherwise no relevance to the study.

Acquisition

Imaging type(s)	Anatomical MRI and functional MRI
Field strength	3T
Sequence & imaging parameters	EPI images were acquired for each participant using a 16-channel head coil and a multiband EPI sequence (TR, 2 s; TE, 30 ms; voxel size, 2.4mm3 isotropic; multiband acceleration factor, 2, 28 slices).<br><br>Quantitative magnetic resonance imaging (qMRI) full-brain anatomical measurements were obtained using the protocols in Mezer et al. (2013) and with a phase-array 32-channel head coil. T1 relaxation times were measured from four spoiled gradient echo (spoiled-GE) images (flip angles: 4°, 10°, 20°, 40°; TR: 14 ms; TE: 2.4 ms; voxel size: 0.8 mm x 0.8 mm x 1.0mm).
Area of acquisition	Slices were aligned parallel to the parieto-occipital sulcus as this is where the visual cortex is.

Diffusion MRI ☐ Used ☒ Not used

Preprocessing

Preprocessing software	fmriPrep v.20.0.1
Normalization	Data were not normalized but were rather analyzed on each participant's native brain surface (fsnative surface) as the study intended to assess individual differences, thus normalization to a standard space was not appropriate.
Normalization template	The data were not normalized
Noise and artifact removal	The pRF analysis detrends the data by projecting out 3 low frequency vectors (the first 3 terms of the discrete cosine transform). There is no other de-noising or artifact removal. Repeated scans with identical stimuli combined with a model based analysis make the paradigm robust to noise
Volume censoring	Data were not censored as there were minimal artifacts; for each participant, their scans and the volumes within were averaged and included as input into the fMRI population receptive field model.

Statistical modeling & inference

Model type and settings	Retinotopic maps were generated using the population receptive field model
Effect(s) tested	Fit of the pRF model to the time-series data (vertex-wise)

Specify type of analysis: ☒ Whole brain ☐ ROI-based ☐ Both

Statistic type for inference (See Eklund et al. 2016)	Population receptive field model was fit to each vertex on the whole brain surface (vertex-wise). We did not do null hypothesis testing of the fMRI data alone.
Correction	Not appropriate for the population receptive field model.

Models & analysis

n/a	Involved in the study
☒	☐ Functional and/or effective connectivity
☒	☐ Graph analysis
☒	☐ Multivariate modeling or predictive analysis
